# Gas migration characteristics in vehicle tank under different ventilation conditions

**DOI:** 10.1038/s41598-023-47772-8

**Published:** 2024-02-02

**Authors:** Yinqing Wang, Chunli Yang, Yan Liu, Xiangchun Li

**Affiliations:** 1 School of Emergency Management and Safety Engineering, China University of Mining and Technology, Beijing, 100083 People’s Republic of China; 2 Occupational Hazards Control Technology Center, Institute of Urban Safety and Environmental Science, Beijing Academy of Science and Technology, Beijing, People’s Republic of China; 3 State Key Laboratory of Explosion Science and Technology, Beijing Institute of Technology, Beijing, 100081 People’s Republic of China

**Keywords:** Mechanical engineering, Energy infrastructure

## Abstract

In recent years, due to the frequent occurrence of accidents in confined space operations, horizontal ammonia tank trucks with higher accident frequencies were selected for numerical simulation research through comparative analysis. The ammonia concentration variation characteristics of horizontal ammonia tank cars were simulated under four conditions: natural ventilation with 0° incoming air, natural ventilation with 45° incoming air, mechanical ventilation with extraction, and mechanical ventilation with compression. The results indicate that natural ventilation requires 48 h to reduce the ammonia concentration to a safe range for operation, while mechanical ventilation reduces the ammonia concentration to infinity and approaches zero within 30 min according to regulations, making the working environment safer; Set up monitoring points inside the tank to monitor the gas disturbance inside the tank at different wind speeds. Based on the ammonia concentration cloud map and the monitoring point wind speed, it can be concluded that local ammonia accumulation is more likely to occur on both sides of the tank due to poor ventilation. Comparing and analyzing the simulated values with theoretical calculations and experiments, it was found that there are differences in the degree of gas change but the overall trend is the same. This indicates that ventilation simulation and the determination of ammonia migration characteristics have practical significance for guiding on-site operations.

## Introduction

In recent years, large production safety accidents such as poisoning and suffocation in confined space operations have occurred frequently, and the safety situation has been complicated and severe, causing heavy losses to the safety of people's lives and properties. Because the limited space is closed or semi-closed for a long time, its entrance and exit are limited and the natural ventilation is poor, it is easy to cause the accumulation of toxic and harmful gases and the low oxygen concentration in the confined space^1,2^. To ensure the safety of workers in confined spaces, it is clearly stipulated in relevant domestic and foreign standards^3–7^, Operators must ventilate the confined space before working, only after the detection values of the concentrations of oxygen, toxic and harmful gases, and flammable and explosive gases in the confined space are qualified, the operators can enter their internal operations^8^. In 2013, the State Administration of Work Safety issued the Interim Regulations on Safety Management and Supervision of Limited Space Operations for Industrial and Trading Enterprises, it is proposed that the operation in confined space should strictly abide by the principle of “ventilation first, inspection later, and operation later”, in the “Five Regulations on Safe Operation in confined Spaces” issued in 2014, re-emphasized that confined space operations must be “ventilated first, then tested, and then operated”, it is strictly forbidden to operate under the condition of ventilation and unqualified testing^9^. Ventilation includes natural ventilation and mechanical ventilation, among them, mechanical ventilation relies on the fan as the power for ventilation, and the wind pressure generated by the high-speed rotation of the fan forces the air in the room to flow to achieve the purpose of ventilation. In the case of insufficient natural ventilation power, mechanical ventilation provides a safe guarantee for operations in confined spaces. Therefore, it is of great significance to study the characteristics of gas transport under mechanical ventilation.

At present, domestic and foreign scholars have conducted useful research on the effect of ventilation on gas transport in confined space, for example, Yang^8^ used numerical simulation method to study the flow field characteristics of single opening confined space under the condition of natural ventilation. Tan et al.^9^ adopted the method of numerical simulation to study the distribution law of the internal flow field, temperature field, oxygen volume fraction and carbon dioxide volume fraction in a confined space under mechanical ventilation. Deng^10^ studied the gas flow characteristics in confined space, especially the indoor space where people live. Feng^11^ studied the natural gas diffusion and explosion flow field in the confined space dominated by pipelines. Nie et al.^12^ studied the diffusion of poisonous gas in confined space integrating environmental factors. Yang^13^ studied the diffusion law of ammonia leakage in a confined space dominated by pipelines. Xie et al.^14^ studied the diffusion law of combustible gas in confined space with obstacles. Shi et al.^15^ studied the distribution law of hydrogen sulfide gas in the sewage pipe network. Xue^16^ studied the diffusion of working fluid leakage of ammonia refrigeration system in confined space. Liu et al.^17^ studied the distribution law of gas concentration in coal mine roadways. He^18^ studied the leakage and diffusion of unsteady gas in indoor natural gas pipelines. Lv and others^19,20^ interpreted the “Technical Specification for Safety in Underground Confined Space Operation Part 2: Gas Detection and Ventilation”. Zhao et al.^21^ studied the migration of natural gas in tight reservoirs. Wei et al.^22^ studied the gas migration law under underground mining conditions. Lu et al.^23^ studied the gas migration law in goaf. Quan and others^24^ studied the ventilation method of cylindrical confined space. Traditional ventilation methods are difficult to remove pollutants in this workplace. Therefore, to ensure the safety of workers, a vortex ventilation model was proposed. The removal effect of pollutants under the action of eddy current is twice that of ordinary ventilation. Pouzou et al.^25^ studied the ventilation effect of confined spaces in shipbuilding enterprises, observed the frequency of welding workers using ventilation, and detected their exposure to particulate matter; In order to study the influence of obstacles, leakage speed, leakage direction and wind speed on the leakage and diffusion of mixed gas. Schmidt^26^ took hydrogen–oxygen mixed gas as the research object, and simulated the leakage and diffusion of mixed gas through FLUENT, and found that the above 4 these indicators have a serious impact on the leakage and diffusion laws of mixed gases. Then Sklavounos^27^ took LNG and oxygen as the research objects, simulated their diffusion through numerical software, and analyzed and summarized the quantitative relationship between the leakage time and the maximum gas concentration. The research results are of great significance. Tauseef^28^ studied the diffusion behavior of heavy gas in the diffusion environment with obstacles and analyzed the fluctuation of gas concentration caused by gravity during the diffusion process. The results found that the Realizable k–s model has higher performance than the standard k–ε model simulation accuracy.

It can be seen from the above studies that scholars have done some research on gas transport in the confined space underground and in confined spaces above ground, most scholars use numerical simulation and other methods to study and analyze the diffusion of different toxic and harmful gases in limited space. According to the different properties of gases, there are different differences in the diffusion speed, height and concentration. And through the simulation of the traditional ventilation mode, cylindrical ventilation, mixed ventilation and other different ventilation mode simulation research; However, there are few studies on gas transport in closed equipment, and there are few studies on gas diffusion in different ways in closed equipment, but there are few types of research on gas transport in closed equipment. Aiming at the gas problem existing in closed equipment, this paper adopts the method of FLUENT numerical simulation to study the gas of closed equipment under mechanical ventilation.

## Closed equipment accident analysis

**Figure 1:**
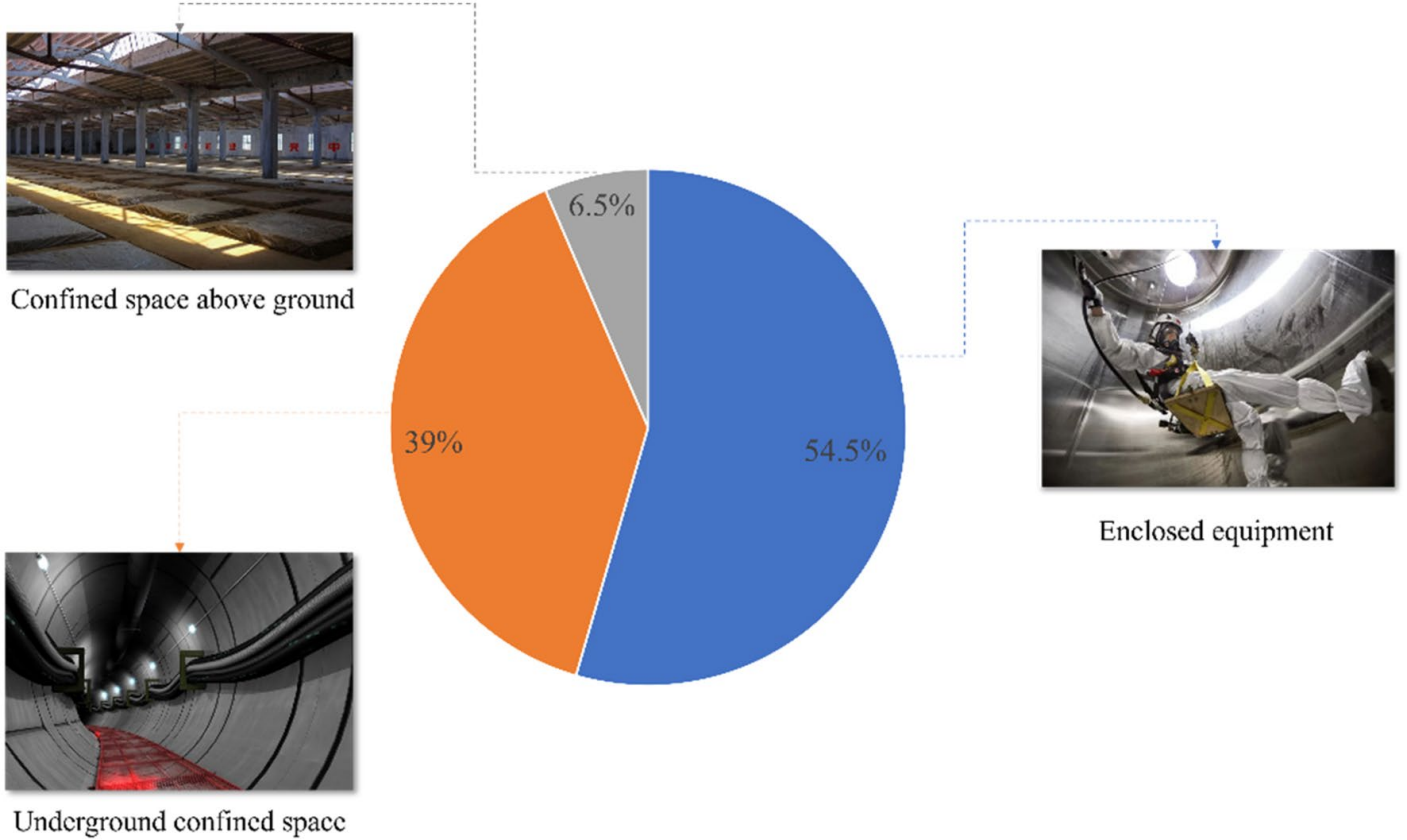
confined space accident statistics. Part of the picture vehicle information comes from the Internet.

Confined space is mainly divided into underground confined space, above-ground confined space, and closed equipment. Among them, the confined space is intended to emphasize the scope of the space and does not mean to be isolated from the outside world, while the closed space emphasizes the complete isolation from the outside world. Closed space is a space with poor ventilation and easy accumulation of toxic and harmful substances. Due to poor ventilation and complex air composition, compared with general workplaces, there are more dangerous and harmful factors, such as oxygen deficiency, the existence of carbon monoxide, hydrogen sulfide, accumulated flammable gases and other harmful gases, dust or smoke, and other hazards. Therefore, this paper analyzes the closed equipment accidents in the past 5 years and counts a total of 42 closed equipment accidents as shown in Fig. 1.

It can be seen from Fig. 1 that the accident probability of closed equipment is greater than 50%. At the same time, it can be obtained through a calculation that among the 77 limited space accidents, the accident probability of closed equipment accounts for 54.5%, and the probability of accidents in underground confined space is about 39%, the probability of an accident in confined space above ground is about 6.5%, and the probability of an accident in closed equipment is greater than that in confined space above ground and confined space underground. Therefore, it is of great significance to study the causes of accidents in closed equipment and reduce the probability of accidents in closed equipment. For the 42 closed equipment accidents that occurred, the frequency of accidents of each type of equipment was also counted as shown in Table 1.

**Table 1:** Frequency of closed equipment accidents.

Closed equipment	Accident frequency
Storage (tank) tank, vehicle-mounted tank	15 times
Cabin	11 times
Pipeline	8 times
Reaction tower (kettle)	4 times
Kiln	2 times
Boiler	2 times

It can be seen from the table that tanks such as storage (tank) tanks and vehicle-mounted tanks are more prone to accidents than other closed equipment. Among the 15 tank accidents, 8 accidents were due to poisoning and suffocation accidents that occurred when maintenance personnel entered tank equipment or tank trucks for maintenance operations and clean-up operations, including suffocation in cleaning oil tankers, and poisoning and suffocation in cleaning and neutralizing tanks, tank car maintenance suffocation, material tank maintenance poisoning, hydrolysis protection tank maintenance poisoning suffocation, oil storage tank maintenance suffocation, poisoning suffocation when cleaning sulfuric acid tank cleaning oil pipe suffocation, etc. There were 4 accidents due to the operator’s maintenance of the storage tank. A flash explosion occurred at the time of operation; there were 3 accidents due to fire and explosion during the operation. For example, on January 11, 2022, in a car wash shop in Anhui Province, when cleaning a tank truck, the driver of the tank truck and two staff members of the car wash shop suffocated, and three people died after 120 ambulance personnel failed to rescue them on the spot. It can be seen that most of the causes of accidents in tanks are poisoning and suffocation. Among the other types of closed equipment accidents, cabin accidents are mainly poisoning and suffocation accidents. There are 9 casualties caused by suffocation, accounting for about 82% of the ship closed equipment accidents; accidents in pipelines are caused by the poor ventilation system, operation personnel poisoning, and suffocation; 75% of reaction tower(kettle) accidents are caused by poisoning and suffocation caused by poor ventilation inside the equipment; 50% of kiln and boiler equipment also cause poisoning and suffocation of workers.

Among the 42 closed equipment accidents in the statistics, 30 closed equipment accidents were caused by poisoning and suffocation due to poor ventilation, accounting for 71% of the total closed equipment accidents; at the same time, in 42 accidents, storage (tank) tanks, vehicle tanks, and other tank accidents reached 15, accounting for 36% of the total closed equipment accidents. The accident types and statistics are based on official accidents. Therefore, this paper selects the tank equipped with the most accidents for research, and mainly simulates the gas flow inside the tank before the staff performs maintenance and cleaning work, to reduce the accident of closed equipment.

## Governing equations for numerical simulation

In computational fluid dynamics, the governing equations for describing multi-component three-dimensional unsteady turbulent flow are established mainly according to the four laws of conservation of mass, conservation of momentum, conservation of energy, and conservation of component transport. Therefore, in this numerical simulation, the basic conservation law should be followed in the numerical simulation settlement process^29^:

1. Mass Conservation Equation.

In describing the fluid flow, the law that should be followed first should be the law of mass conservation. On the one hand, the law of mass conservation is the basic law that mass transfer should obey. On the other hand, in the process of fluid flow, no matter what flows from the fluid, the total mass of the flowing fluid should be constant. The simulated fluid is a steady flow and in compressible fluid, the density of the fluid is a constant, and the law of conservation of mass is expressed as^30^:

1 $$\frac{{\partial \left( {\rho u} \right)}}{\partial x} + \frac{{\partial \left( {\rho v} \right)}}{\partial y} + \frac{{\partial \left( {\rho w} \right)}}{\partial z} = 0$$

The meaning of each parameter is: ρ represents fluid density; *t* represents time; u, v, and w represent the projection of velocity vector in x, y, and z directions, respectively.

(2) Momentum conservation equation.

The meaning described by the law of conservation of momentum means that the action of the external force on the unit fluid micro-element will cause the fluid micro-element to generate momentum, and the momentum of the fluid micro-element is equal to the rate of change of the momentum of the fluid with time, This explanation is often referred to as Newton’s second law, and in the equations expressed, this equation is also often referred to as the N–S equation^31^:

2 $$\left\{ \begin{gathered} \frac{{\partial \left( {\rho uu} \right)}}{\partial x} + \frac{{\partial \left( {\rho uv} \right)}}{\partial y} + \frac{{\partial \left( {\rho uw} \right)}}{\partial z} = \frac{\partial }{\partial x}\left( {\mu \frac{\partial u}{{\partial x}}} \right) + \frac{\partial }{\partial y}\left( {\mu \frac{\partial u}{{\partial y}}} \right) + \frac{\partial }{\partial z}\left( {\mu \frac{\partial u}{{\partial z}}} \right) - \frac{\partial p}{{\partial x}} + S_{u} \hfill \\ \frac{{\partial \left( {\rho vu} \right)}}{\partial x} + \frac{{\partial \left( {\rho vv} \right)}}{\partial y} + \frac{{\partial \left( {\rho vw} \right)}}{\partial z} = \frac{\partial }{\partial x}\left( {\mu \frac{\partial v}{{\partial x}}} \right) + \frac{\partial }{\partial y}\left( {\mu \frac{\partial v}{{\partial y}}} \right) + \frac{\partial }{\partial z}\left( {\mu \frac{\partial v}{{\partial z}}} \right) - \frac{\partial p}{{\partial y}} + S_{v} \hfill \\ \frac{{\partial \left( {\rho wu} \right)}}{\partial x} + \frac{{\partial \left( {\rho wv} \right)}}{\partial y} + \frac{{\partial \left( {\rho ww} \right)}}{\partial z} = \frac{\partial }{\partial x}\left( {\mu \frac{\partial w}{{\partial x}}} \right) + \frac{\partial }{\partial y}\left( {\mu \frac{\partial w}{{\partial y}}} \right) + \frac{\partial }{\partial z}\left( {\mu \frac{\partial w}{{\partial z}}} \right) - \frac{\partial p}{{\partial z}} + S_{w} \hfill \\ \end{gathered} \right\}$$

In the formula:ρ means density; *u*, *v*, *w* mean the projection of velocity vector in *x*, *y*, *z* directions respectively; μ means dynamic viscosity coefficient of fluid; p means unit fluid micro-element The pressure of the shared body; the meanings of Su, Sv, Sw are the generalized source terms of the momentum conservation equation.

(3) Component mass conservation equation.

Another control equation that is mainly used in this numerical simulation experiment is the component transport equation, which mainly controls the change and hold of various gas concentrations during the transport of gas components in the porous medium. According to the description, the component mass conservation equation can be expressed as^32^:

3 $$\frac{{\partial \left( {\rho c_{s} } \right)}}{\partial t} + div(\rho Uc_{s} ) = div(D_{s} grad(\rho c_{s} )) + S_{u} + R_{s}$$

In the formula: c_s_ means the volume concentration of the component S; ρc_s_ means the mass concentration of the component volume; Ds means the component diffusion coefficient; Ss means the component generation rate in the micro element.

(4) Energy conservation equation.

The meaning expressed by this law is that the increased rate of energy in the micro-unit is equal to the net heat entering the micro-unit plus the work done by the physical force and the surface force on the micro-unit. Its expression^33^:

4 $$\frac{{\partial \left( {\rho T} \right)}}{\partial t} + div(\rho uT) = div\left( {\frac{{k{\prime} }}{{c_{p} }}gradT} \right) + S_{r}$$

In the formula: T represents the ambient temperature; k' represents the heat transfer coefficient of the fluid; cp means the specific heat capacity of the fluid; Sr means the heat source in the fluid and the heat generated by the viscous action during the fluid flow; other symbols have the same meanings as above. In fluid mechanics, the Schmidt number (Sc) is a dimensionless scalar, defined as the ratio of the motion viscosity coefficient to the diffusion coefficient, used to describe a fluid with both momentum diffusion and mass diffusion, and is physically related to the relative thickness of the hydrodynamic layer and the mass transfer boundary layer.

## Simulation scenarios and computational models

### Simulation program

#### Basic assumptions

Since it is very difficult to fully simulate the transport characteristics of ammonia gas, to facilitate the numerical simulation of the transport characteristics of ammonia gas, the following assumptions are made^34^:

1. The gases involved in the tank are all ideal gases;
2. The ambient temperature around the storage tank is the same as the initial temperature inside the storage tank;
3. No phase transition or chemical reaction occurs during the simulation;
4. The acceleration of gravity is constant and does not change with the change of height;
5. After the tanker unloads liquid ammonia, it is at normal pressure, so the pressure environment is standard atmospheric pressure.

#### Simulation scheme

(1) Initial concentration of ammonia gas.

The maintenance of the ammonia tank requires access to the inside of the equipment. Before maintenance, all the medium in the tank is emptied and replaced with gas^35^. Since liquid ammonia is extremely easy to vaporize, after unloading the liquid ammonia inside the tanker, the remaining liquid ammonia in the tank cannot be completely removed. Liquid ammonia volatilizes into gas after a while. Liquid ammonia evaporates very quickly at standard atmospheric pressure, assuming that the ammonia inside the tank is gaseous and the volume concentration of ammonia is 100%^36^.

(2) Ventilation method.

This simulation selects natural ventilation and mechanical ventilation. According to the provisions of “Technical Specification for Safety in Underground Confined Space Operation Part 2: Gas Detection and Ventilation”, the natural ventilation time should be 30 min; the light wind speed should be 1.6–3.3 m/s to simulate. In mechanical ventilation, an axial flow fan is generally placed on the manhole to speed up the gas flow rate, and the fan speed is 10 m/s^37^. The specific ventilation methods are shown in Table 2.

**Table 2:** Ventilation mode setting.

Ventilation	Ventilation rate (m/s)	Ventilation direction	Ventilation ingredients
Natural ventilation	3	0 degree wind (X-axis negative)	21% oxygen and 79% nitrogen
		45-degree wind	
Mechanical ventilation	10	Extraction	
		Press-in	

### Geometric model

**Figure 2:**
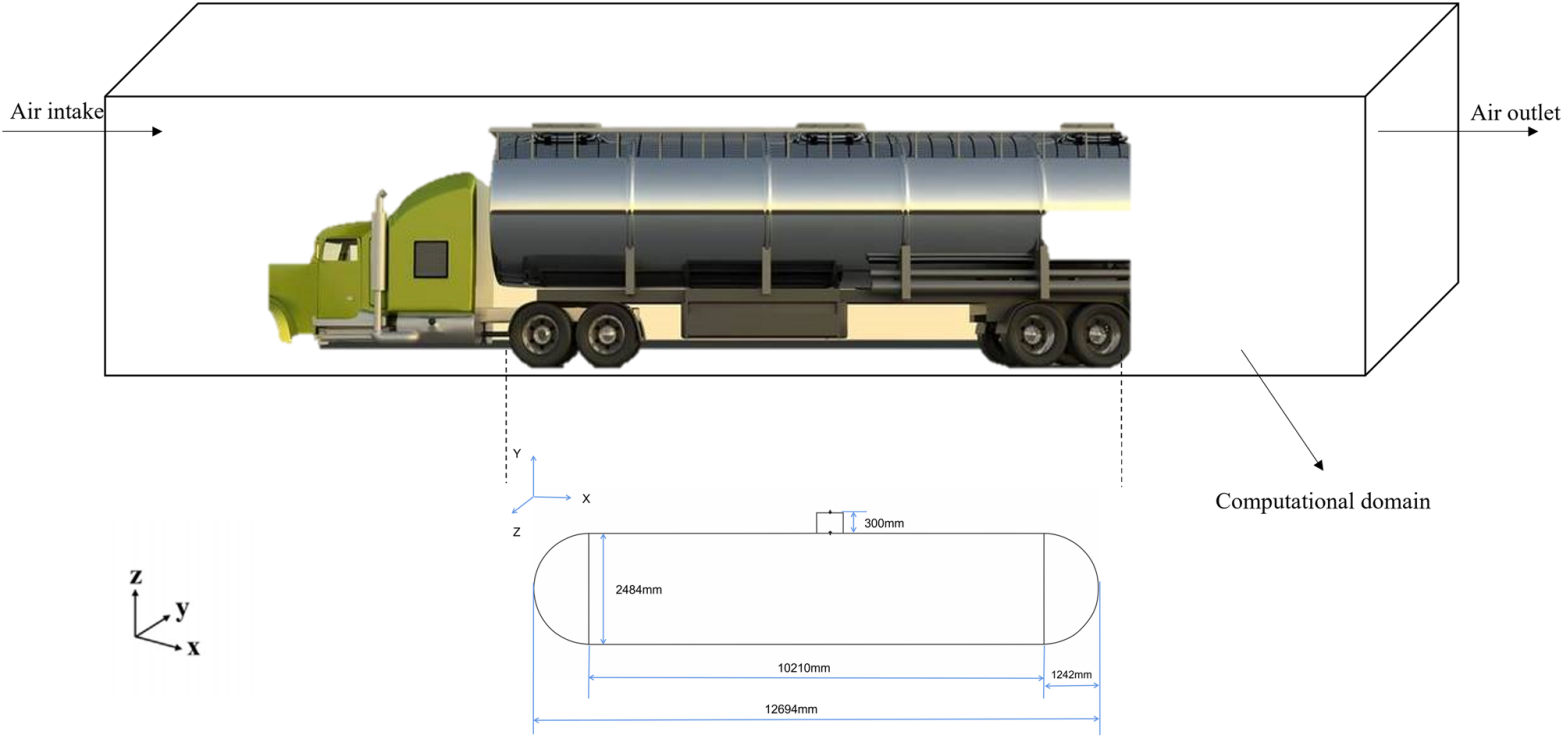
Horizontal tank column tank model. Part of the picture vehicle information comes from the Internet.

This simulation selected a horizontal tank car filled with ammonia gas with an inner diameter of 2484 mm, a length of 10210 mm^37^, and a total length of 12694 mm as the research object. There is a manhole with a height of 300 mm in the center above the horizontal tank for maintenance by staff. The simulated horizontal groove cylindrical tank body is shown in Fig. 2. The simulated size of the entire horizontal tank body is the actual tank body size. According to the size of the horizontal tank car, the calculation domain size under natural ventilation conditions is specified to be 20,000 × 6000 × 8000 mm.

### Mesh size and boundary conditions

The simulation was conducted using FLUENT software. FLUENT software includes pressure-based separation solver, density-based implicit solver and density-based explicit solver. The multi-solver technology enables FLUENT software to simulate various complex flow fields ranging from incompressible to hypersonic. FLUENT software contains very rich and engineering confirmed physical models. Due to the adoption of a variety of solution methods and multi-grid accelerated convergence technology, FLUENT can achieve the best convergence speed and solution accuracy. Flexible unstructured mesh and solve-based adaptive mesh technology and mature physical models can simulate hypersonic flow fields, heat transfer and phase transformation, chemical reactions and combustion, multiphase flow, rotating machinery, dynamic/deformed mesh, noise, material processing and other complex mechanism flow problems. The grid size in numerical simulation has a great influence on the simulation accuracy and computational efficiency^38^. In general, the denser the grid spacing, the more accurate the calculation results long. Therefore, it is necessary to determine the appropriate mesh size for specific problems during simulation calculation and to reduce the number of meshes as much as possible while ensuring the accuracy of numerical simulation, thereby improving the calculation efficiency. In the unsteady simulation, the maximum time step is set to 20 and the number of iterations is set to 150.

**Figure 3:**
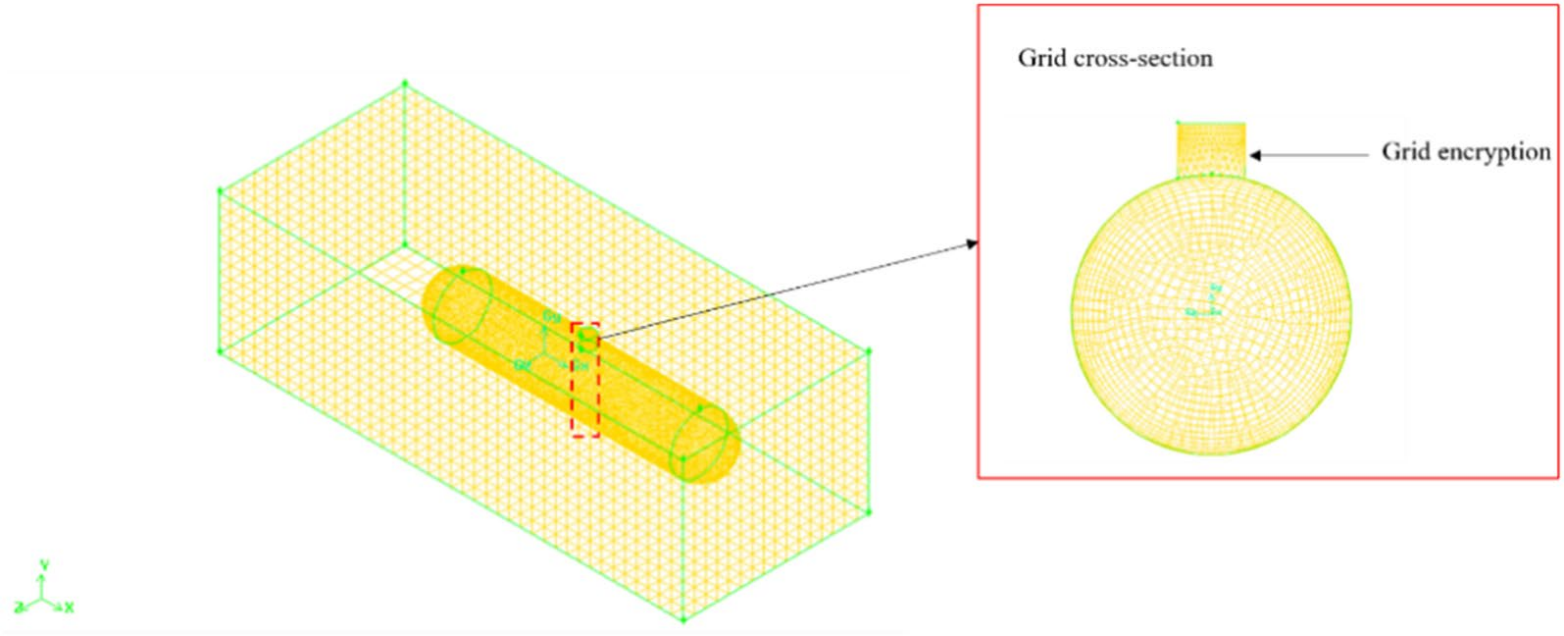
Grid division of computational domain (with tank body).

In this simulation, mesh the opposite side first. Use a Map partition with an interval of 0.5 m on the surface of the computational domain (omitting this step under mechanical ventilation conditions), and use a Pave partition with an interval of 0.01 m on the entire cylindrical surface. Because the manhole requires continuous ventilation, the surface at the manhole is encrypted using a Pave partition with an interval of 0.05 m; Next, the computational domain is divided using TGrid with an interval of 0.5 m (omitted under mechanical ventilation conditions), and the entire horizontal tank body is meshed using TGrid with an interval of 0.01 m. Among them, the 0.01 m grid has 1,213,789 mesh numbers, and the minimum mesh area is 2.379e−8 square meters. The 0.05 m grid has a total of 35,750 mesh numbers, and the minimum mesh area is 1.89e10−6 square meters. The 0.1 m grid has 11,076 mesh numbers, and the minimum mesh area is 5.148e−6 square meters. The 0.2 m grid has 4348 mesh numbers, and the minimum mesh area is 9.588e−5 square meters. Through verification, it is concluded that the denser the grid, the slower the propagation, but in order to enhance the accuracy of the experimental results, the denser grid is selected for simulation. The divided calculation domain (with tank body) is shown in Fig. 3. Because it mainly simulates the gas flow characteristics inside the tank, especially in the case of mechanical ventilation, when the fan is connected, the main situation inside the tank is observed, so the calculation domain is slightly larger than the storage tank.

**Figure 4:**
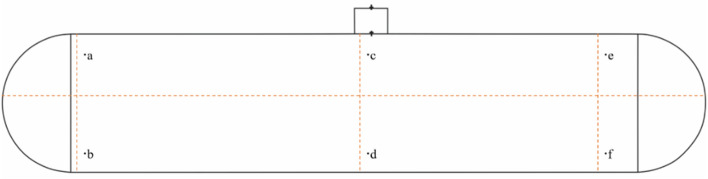
Tank monitoring points.

Set initial boundary conditions in GAMBIT. Under natural ventilation conditions, the left boundary of the calculation domain is the VELOCITY-INLET (velocity inlet) condition, the right boundary of the calculation domain is the OUTFLOW (free flow) condition, the boundary at the manhole is the INTERFACE (internal interface) condition, and the remaining calculation domain surfaces and tank wall conditions are the WALL (fixed wall) condition. Under mechanical ventilation conditions, the boundary of the extraction manhole is PRESSURE-OUTLET (pressure outlet) condition, while the other tank wall conditions are WALL (fixed wall) condition; The boundary of the pressurized manhole is the VELOCITY-INLET condition, while the remaining tank wall conditions are the wall condition. At the same time, monitoring points are set up inside the tank body, as shown in Fig. 4.

## Simulation results and analysis

Import the model built by Gambit in Fluent, then define the material, select ammonia gas (NH_3_) in the material library that comes with Fluent, read the ammonia gas data directly, and then perform model settings and solver settings, as shown in Table 3.

**Table 3:** Model settings and solver settings.

Model	Solver	Pressure implicit unsteady
	Viscous model	Realizable K-epsilon
	Species	Species transport
	Energy	On
Boundary conditions	Inlet boundary type	Velocity-inlet
		Outflow
		Interface
	Wall	No Slip
Operating conditions	Operating pressure (pascal)	101,325
	Gravitational acceleration	Y =−9.81
Solution controls	Pressure–velocity Coupling	SIMPLE
	Discretization	First Order Upwind
	Convergence criterion	0.00001
Patch	Value	NH3(1)

The turbulent flow diameter D in the parameter setting is based on the formula:

5 $$D = \frac{4A}{S}$$

In the formula, A is the cross-sectional area of the manhole, and S is the perimeter of the manhole; the turbulent flow diameter is 0.6 m by bringing in the data.

The turbulence intensity I in the parameter setting is based on the formula:

6 $$I = 0.16 \times \mathop {\left( {R{}_{eH}} \right)}\nolimits^{{ - \frac{1}{\begin{subarray}{l} 8 \\ \end{subarray} }}}$$

7 $$R{}_{eH} = \frac{Vd}{{1.5 \times 10^{{_{ - 6} }} m^{2} /s}}$$

where V is the velocity, d is the diameter of the manhole, and ReH is the Reynolds number; the turbulence intensity is 4.9% by bringing in the data.

According to the “Safety Technical Specification for Underground Confined Space Operation Part 2: Gas Detection and Ventilation”, it can be seen from the table that the upper limit of the concentration of ammonia gas in the limited space is 9 mg/m^3^, and the density of ammonia gas is 0.7081 g/L, that is, 708.1 mg/m^3^, through the ratio, the upper limit percentage of the highest ammonia concentration is 1.27%, that is, when the highest ammonia concentration is lower than 0.0127, the ventilation can be stopped.

### Simulation results of natural ventilation 0° incoming wind

**Figure 5:**
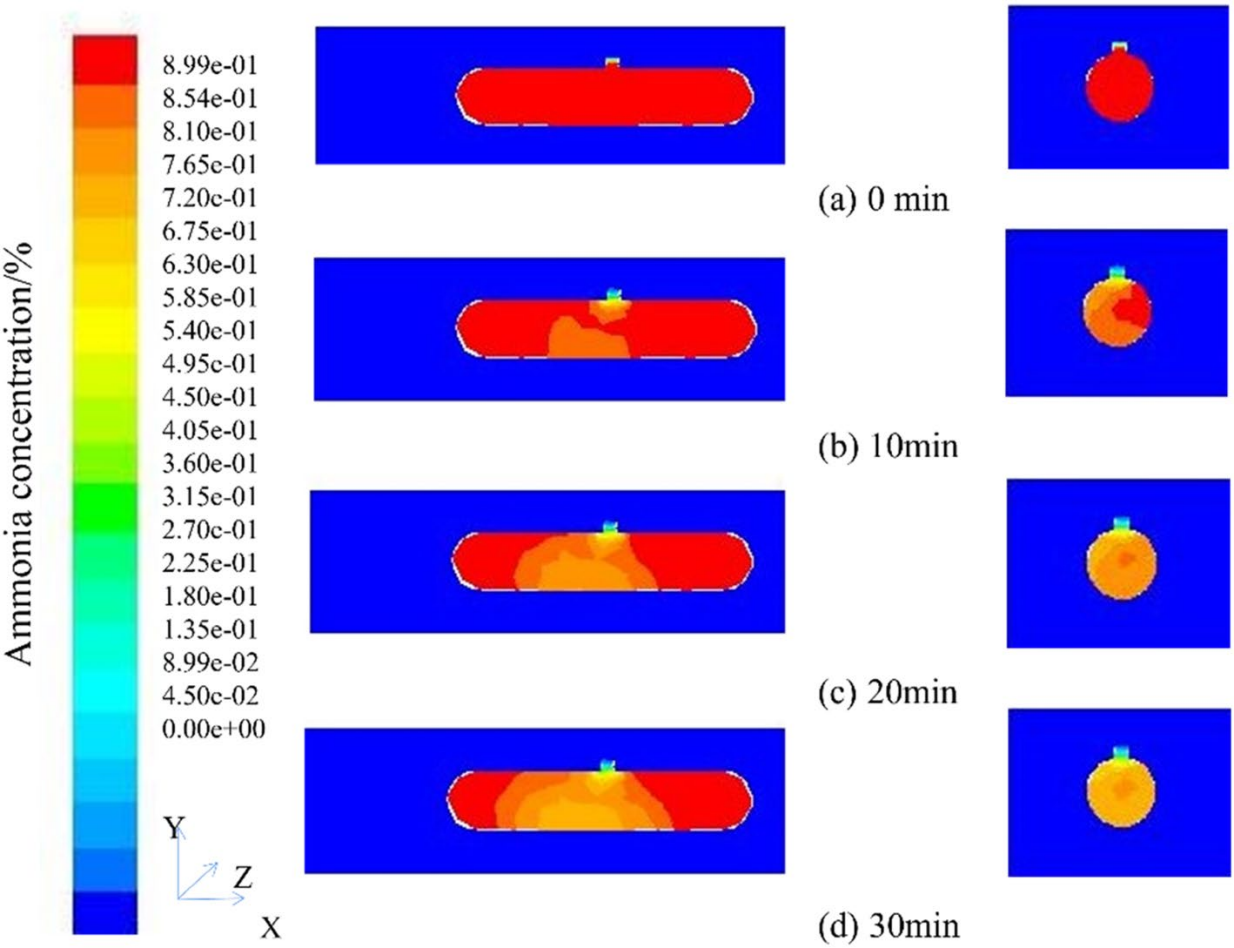
Cloud figure of ammonia concentration distribution at different times (Face and manhole profile).

The cloud figure of ammonia gas concentration distribution of natural ventilation 0° incoming wind at different times (t = 10 min, t = 20 min, t = 30 min) is shown in Fig. 5. To observe the migration characteristics of ammonia gas in the tank easily, the front-view longitudinal section and manhole longitudinal section of the tank and computational domain were selected as the observation objects, and the characteristics of ammonia gas migration were analyzed. The coordinates of the two longitudinal sections are (− 10, 4, − 0.2), (10, 4, − 0.2), (10, − 4, − 0.2); (2.835, 4, − 3), (2.835, 4, 3), (2.835, − 4, 3).

**Figure 6:**
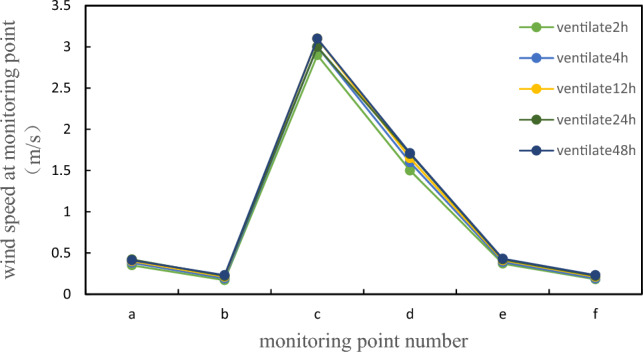
Location figure of ventilation rate monitoring points.

By comparing the concentration cloud maps of the vertical profile from the beginning of ventilation to 30 min of ventilation, it was found that the ventilation volume near the manhole was relatively high, and the diffusion rate of ammonia concentration was fast. The concentration cloud map showed significant changes, with a concentration decrease of about 4.5% every 10 min; The air intake on both sides of the tank body is relatively small, and the diffusion speed of ammonia concentration is slow. The concentration cloud map does not show significant changes. Comparing the concentration cloud map of the vertical profile of the manhole at different times, due to the high ventilation volume, the concentration cloud map shows significant changes, with a concentration decrease of about 8.9% every 10 min. During the entire simulation process, the natural wind speed is 3 m/s and the rate is constant. The overall wind speed change can be measured through monitoring points, as shown in Fig. 6.

After processing the simulation results in FLUENT, the corresponding wind speeds of each detection point were obtained. From the figure, it can be seen that under this ventilation condition without pipeline layout, the wind speed fluctuation amplitude of each detection point is relatively large. The maximum wind speed at detection point c is 3.1 m/s, while the wind speed at detection points a, b, e, and f fluctuates within the range of 0–0.5 m/s. The wind speed at detection point d fluctuates within the range of 1.4–1.8 m/s, and the difference between the peak and minimum wind speeds at detection points is significant, The distribution of wind speed inside the tank is uneven, and after fresh air enters the tank, it is limited to the area near point c opposite the air supply outlet. The gas flow in this area is good, and the wind speed is relatively high, while the gas in other areas has almost no flow. Under normal ventilation conditions, the uniformity of wind speed distribution inside the tank is not ideal, and there is a disadvantage of a dead corner area for air supply. Fresh air cannot effectively flow inside the tank, which is not conducive to diluting the toxic gas in the entire area of the tank. According to Bernoulli’s equation:

8 $$p1 + \frac{1}{2}\rho v1^{2} + \rho gh1 = c$$

It can be seen that during the fluid movement, the pressure changes with the fluid velocity and the height difference. C is a constant, so when the flow rate increases or the height difference increases, the pressure decreases; when the flow rate decreases or the high-pressure difference decreases the pressure increases. The fluid moving at a low speed can be regarded as an in compressible fluid^39^. When the external wind speed hits the outer wall of the tank at 3 m/s, a high-pressure air curtain is formed due to the obstruction, so the airflow at the tank mouth spreads radially to the surrounding^40^; When the pressure increases, the height difference remains unchanged, the flow rate decreases, the flow rate entering the tank decreases, and the diffusion efficiency of ammonia gas decreases.

**Figure 7:**
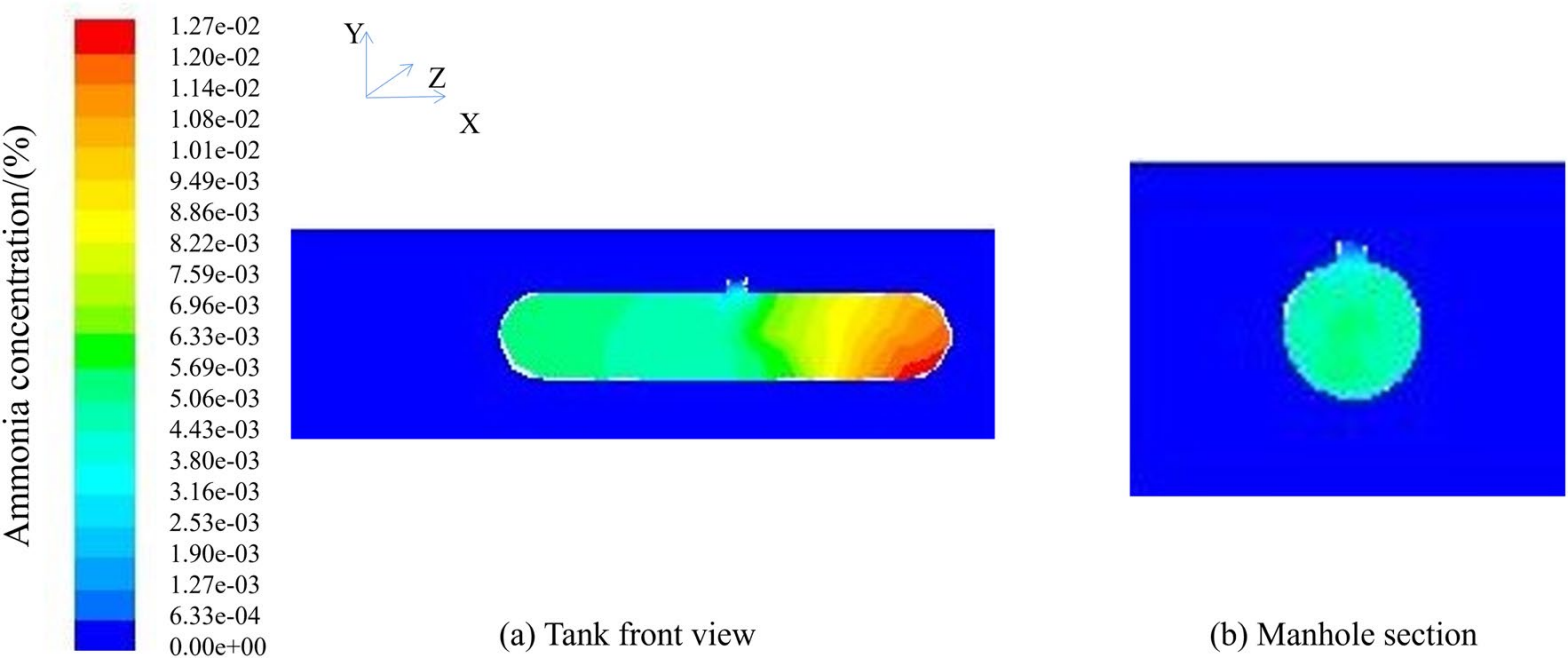
Natural ventilation 0° ventilation 48 h; (**a**) Tank front view; (**b**) Manhole section.

After 30 min of ventilation, the maximum concentration of ammonia still did not drop to the specified minimum concentration of ammonia in the confined space of 0.0127 mg/m^3^, and it was still necessary to continue ventilation. The subsequent ventilation results are shown in Fig. 7.

To reduce the maximum concentration to a safe working range, continue the ventilation simulation until the requirement is reached for 48 h, and then end the simulation. According to the cloud figure, the ammonia concentration in the center of the manhole is lower, and the concentration is higher when going to both sides of the tank. Although the overall maximum concentration is within the safe range, it is easy to cause the local concentration of ammonia gas at both ends of the tank to be too high, causing harm to operators. Under the conditions of natural ventilation of 0° and wind speed of 3 m/s, it takes 48 h to enter the tank before entering the tank.

### Simulation results of natural ventilation 45° incoming wind

**Figure 8:**
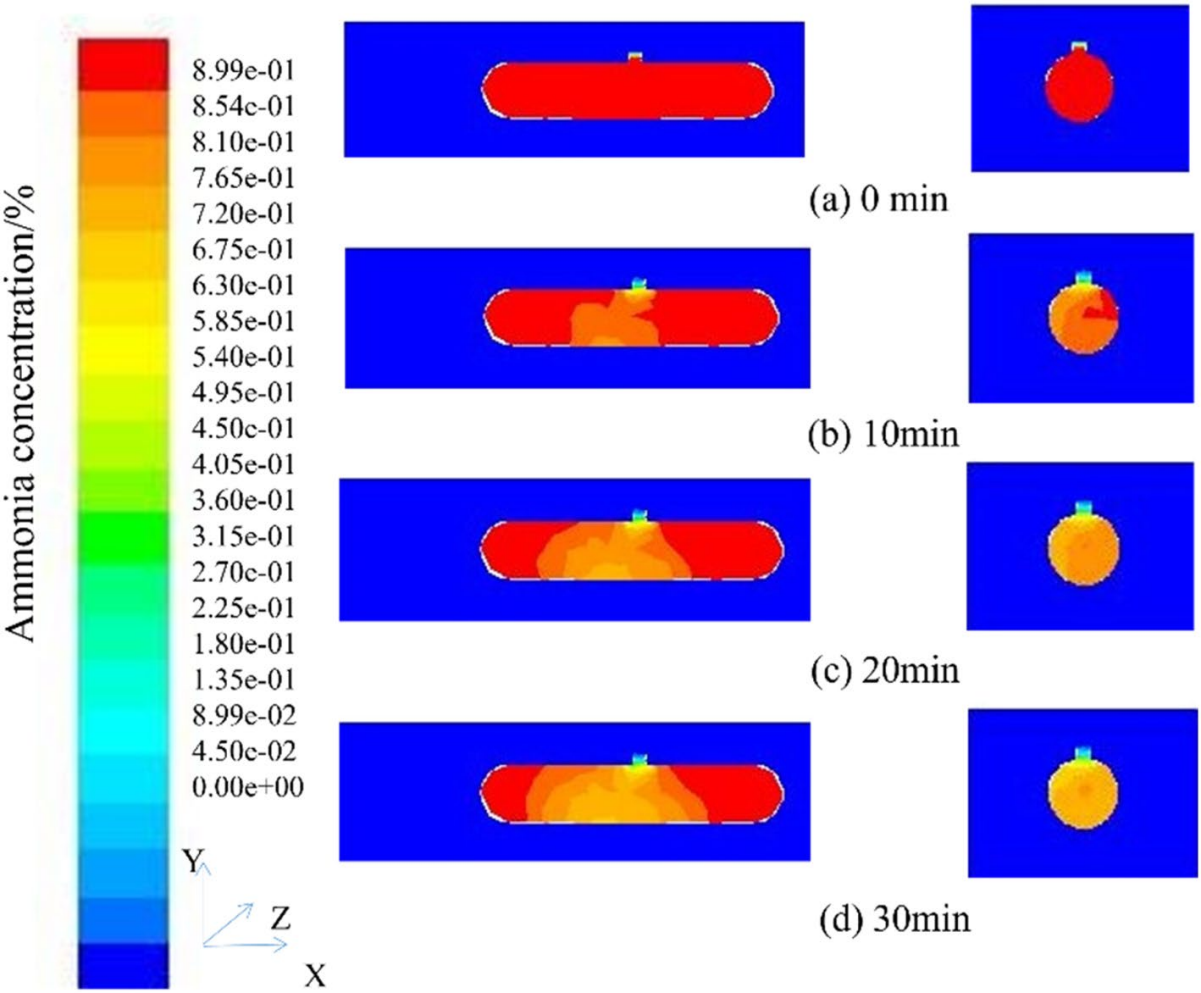
Cloud figure of ammonia concentration distribution at different times (Face and manhole profile).

Figure 8 for the cloud figure of ammonia gas concentration distribution of natural ventilation 45° incoming wind at different times (t = 10 min, t = 20 min, t = 30 min). To observe the migration characteristics of ammonia gas in the tank easily, the front-view longitudinal section and manhole longitudinal section of the tank and computational domain were selected as the observation objects, and the characteristics of ammonia gas migration were analyzed. The coordinates of the two longitudinal sections are (− 10, 4, − 0.2), (10, 4, − 0.2), (10, − 4, − 0.2); (2.835, 4, − 3), (2.835, 4, 3), (2.835, − 4, 3).

By comparing the concentration cloud figure of the vertical profile from the start of ventilation to 30 min of ventilation, the ventilation volume near the manhole is larger, the diffusion rate of ammonia gas concentration is faster, and the concentration cloud figure has obvious changes, and the concentration decreases by about 8.9% every 10 min, compared with the incoming wind at 0°, the concentration decreased by about 4.4%; the air intake on both sides of the tank was small, the diffusion rate of ammonia concentration was slow, and the concentration cloud figure did not change significantly. Comparing the concentration cloud figure of the longitudinal section of the manhole at different times, due to the large ventilation volume, the concentration cloud figure has obvious changes, and the concentration decreases by about 8.9% every 10 min, which is the same as the 0° wind diffusion speed.

During the entire simulation process, the natural wind speed is 3 m/s and the rate is constant. The overall wind speed change can be measured through monitoring points. Similarly, as shown in Fig. 7, after fresh air enters the tank, it is limited to the area near point c opposite the air supply outlet. This area has good gas flow and high wind speed, while the gas in other areas has almost no flow. The uniformity of wind speed distribution in the tank under normal ventilation conditions is not ideal, There is a disadvantage in the dead corner area of air supply, where fresh air cannot flow effectively within the tank, which is not conducive to diluting the toxic gases in the entire area of the tank.

The outside wind direction is inclined at 45° to the ground, and the wind speed is constant at 3 m/s. The wind speed near the front end of the tank and the entire lower end of the tank is lower than 3 m/s, and the wind speed at the upper end of the tank recovers to 3 m/s; the wind speed decreases significantly after entering the tank, the average wind speed inside is less than 1 m/s, so it also hinders the diffusion of ammonia concentration. Similarly, according to Eq. (8), it can be concluded that the airflow at the mouth of the air tank diffuses rapidly to the surroundings in a radial manner, and the flow rate entering the tank decreases, and the diffusion efficiency of ammonia decreases.

**Figure 9:**
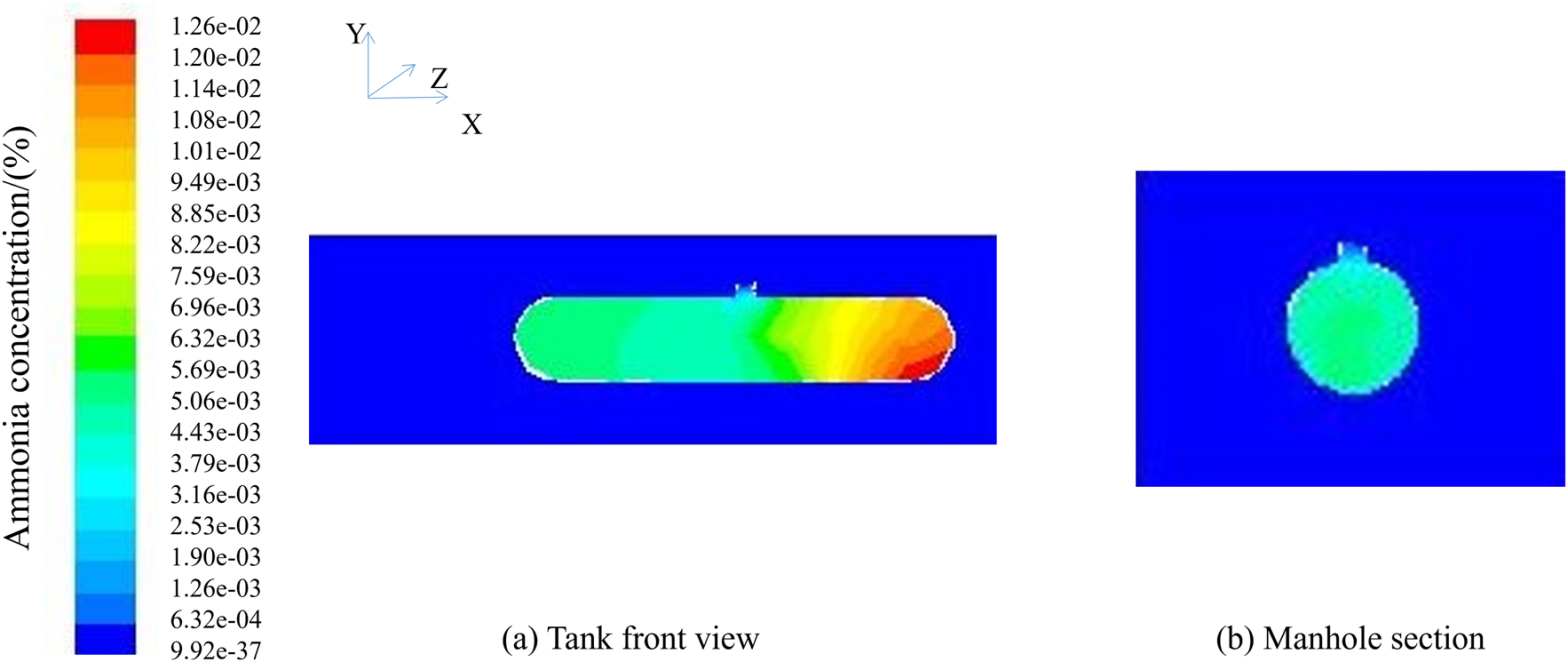
Natural ventilation 45° ventilation 48 h; (**a**) Tank front view; (**b**) Manhole section.

After 30 min of ventilation, the maximum concentration of ammonia still did not drop to the specified minimum concentration of ammonia in the limited space of 0.0127 mg/m^3^, and it was still necessary to continue ventilation. The subsequent ventilation results are shown in Fig. 9.

To reduce the maximum concentration to a safe working range, continue the ventilation simulation until the requirement is reached for 48 h, and then end the simulation. According to the cloud figure, the ammonia concentration in the center of the manhole is lower, and the concentration is higher when going to both sides of the tank. Although the overall maximum concentration is within the safe range, it is easy to cause the local concentration of ammonia gas at both ends of the tank to be too high, causing harm to operators. Under the conditions of natural ventilation of 45° and wind speed of 3 m/s, it takes 48 h to enter the tank before entering the tank.

### Simulation results of the mechanical ventilation extraction

**Figure 10:**
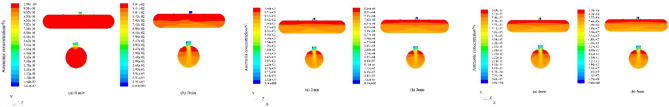
Cloud figure of ammonia concentration distribution at different times (Face and manhole profile).

The concentration distribution cloud figure of ammonia gas extracted by mechanical ventilation at different times (t = 1 min, t = 2 min, t = 3 min, t = 4 min, t = 5 min) is shown in Fig. 10. To observe the migration characteristics of ammonia gas in the tank easily, the front view of the tank body and the section at the manhole was selected as the observation objects to analyze the migration characteristics of ammonia gas. The selected coordinates of the section at the manhole are (5.4, 0, 0), (5.4, 1.7, 0), (5.4, 1.7, 1.242).

By comparing the concentration cloud diagrams from 1 to 5 min of ventilation, the ammonia concentration changed significantly during 1–2 min of ventilation, which decreased by 9.1%, and the concentration of ammonia decreased by 5.8% for 2–3 min of ventilation. The concentration of ammonia gas decreased by 5.8% in 4 min, and the concentration of ammonia gas decreased by 5.7% in 4–5 min of ventilation. In the front view of the tank, the concentration of ammonia diffuses rapidly along the Y-axis at the manhole, and the concentration is low. The concentration of ammonia gradually increases as it goes to both sides of the tank, and it is difficult to diffuse out; in the cross-sectional view of the manhole, the ammonia concentration at the manhole and the downward tank section is lower, and the ammonia concentration gradually increases as it goes to both sides of the tank wall.

**Figure 11:**
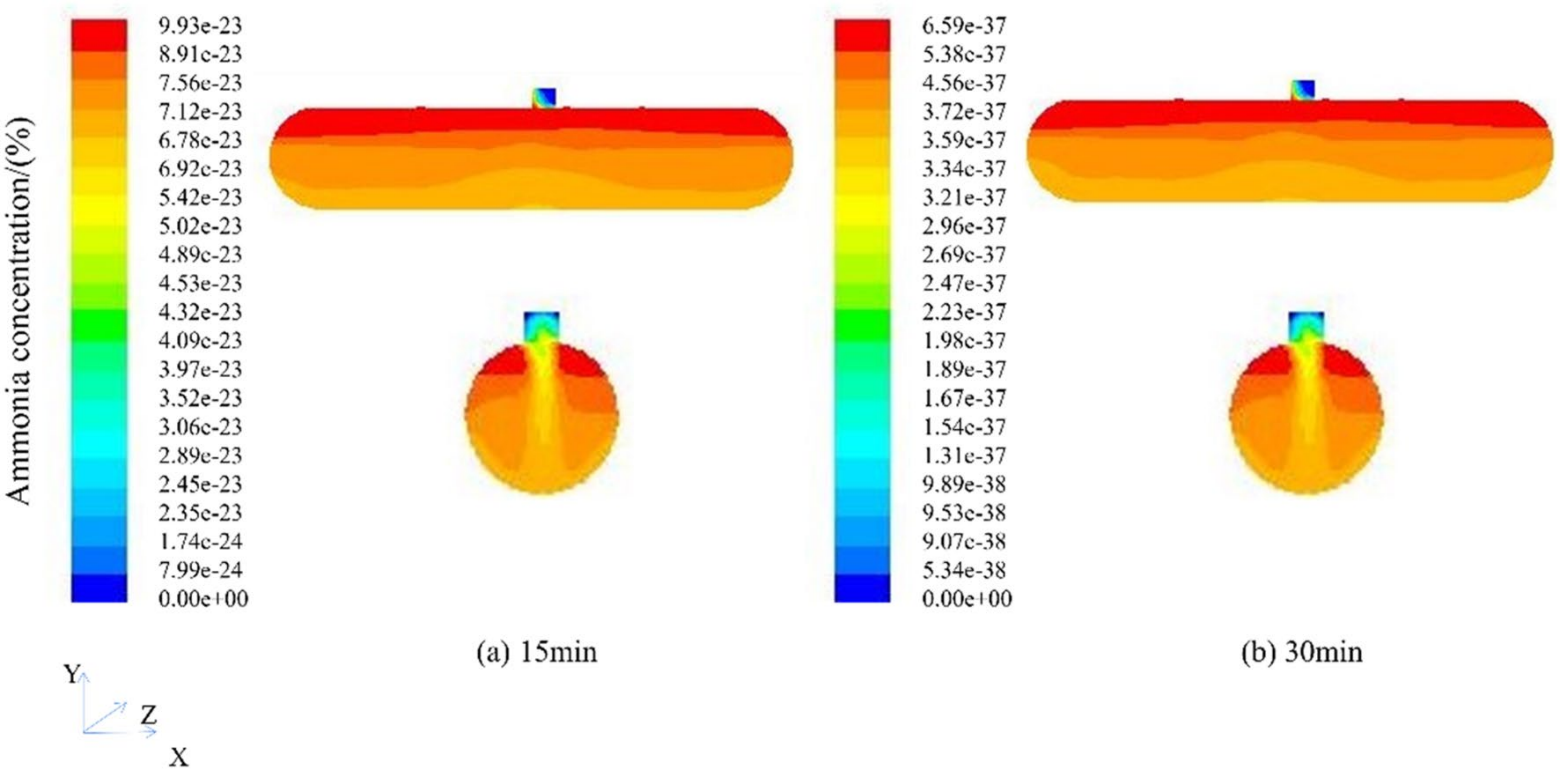
Cloud figure of ammonia concentration distribution at different times (Face and manhole profile).

According to the cloud figure of ammonia gas concentration of the mechanical extraction type, under the condition of a ventilation rate of 10 m/s, ventilation can be stopped for 2 min. However, in the actual operation process, to prevent the local ammonia concentration from being too high, a 5-min ammonia concentration distribution cloud figure was simulated. After 5 min of ventilation, the highest ammonia concentration in the tank was 5.78e−06, which can be carried out as Repair work. According to the principle of confined space detection, the detection time should not be earlier than 30 min before the start of the operation and continue to simulate until 30 min. The obtained ammonia concentration distribution cloud figure is shown in Fig. 11.

**Figure 12:**
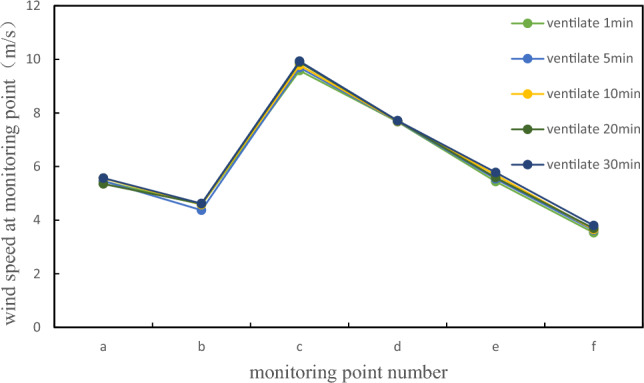
Ammonia gas rate for 5 min of ventilation;

Ventilation to 30 min, the highest concentration of ammonia in the tank is 6.59e−37, and the concentration is infinitely close to 0, which meets the maintenance conditions and meets the maintenance principle, and can be relatively safe for cleaning and maintenance operations. To ensure the safety of the operators, even if the maintenance standards are met, to prevent the local concentration from being too high in the actual process, the operators should be equipped with operation equipment that has passed the quality inspection. Due to the mechanical ventilation rate set at the manhole being 10 m/s, the overall wind speed change can be measured through monitoring points, as shown in Fig. 12.

After processing the simulation results in FLUENT, the corresponding wind speeds at each detection point were obtained. From the figure, it can be seen that under this mechanical ventilation condition, the wind speed fluctuation at each detection point is relatively small, and the overall wind speed is relatively large. At detection point c, the maximum wind speed is 9.93 m/s, while at detection points a, b, e, and f, the wind speed fluctuates within the range of 3–6 m/s. At detection point d, the wind speed fluctuates within the range of 7–8 m/s. The difference between the peak and minimum wind speeds at detection point d is relatively small, and the wind speed distribution inside the tank is relatively uniform. After fresh air enters the tank, it is limited to the area near point c opposite the air supply outlet, which has good gas flow and high wind speed, Compared to natural ventilation, the gas in other areas has fluidity, and the uniformity of wind speed distribution in mechanically ventilated tanks is relatively ideal. Although there is a disadvantage of blind air supply areas, fresh air cannot effectively flow inside the tank, which is not conducive to diluting the toxic gas in the entire area of the tank, the overall ventilation efficiency is much higher than that of natural ventilation.

### Mechanical ventilation press-in simulation results

**Figure 13:**
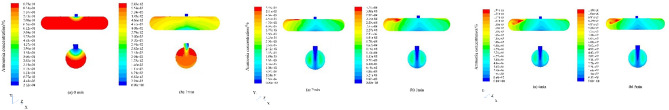
Cloud figure of ammonia concentration distribution at different times (Face and manhole profile).

The cloud figure of the concentration distribution of ammonia gas in the mechanical ventilation press-in type at different times (t = 1 min, t = 2 min, t = 3 min, t = 4 min, t = 5 min) is shown in Fig. 13. To observe the migration characteristics of ammonia gas in the tank easily, the front view of the tank body and the section at the manhole was selected as the observation objects to analyze the migration characteristics of ammonia gas. The selected coordinates of the section at the manhole are (5.4, 0, 0), (5.4, 1.7, 0), (5.4, 1.7, 1.242).

By comparing the concentration cloud diagrams from 1 to 5 min of ventilation, the ammonia concentration changed significantly during 1–2 min of ventilation, which decreased by 9.3%, and the concentration of ammonia decreased by 6% for 2–3 min of ventilation. The extraction type increased by 0.2%, the ammonia gas concentration decreased by 5.9% for 3–4 min of ventilation, and the ammonia gas concentration decreased by 5.8% for 4–5 min of ventilation, which was 0.1% higher than that of the extraction type. In the front view of the tank, the concentration of ammonia diffuses rapidly along the Y-axis at the manhole, and the concentration is low. The concentration of ammonia gradually increases as it goes to both sides of the tank, and it is difficult to diffuse out; in the cross-sectional view of the manhole, the ammonia concentration at the manhole and the downward tank section is lower, and the ammonia concentration gradually increases as it goes to both sides of the tank wall.

**Figure 14:**
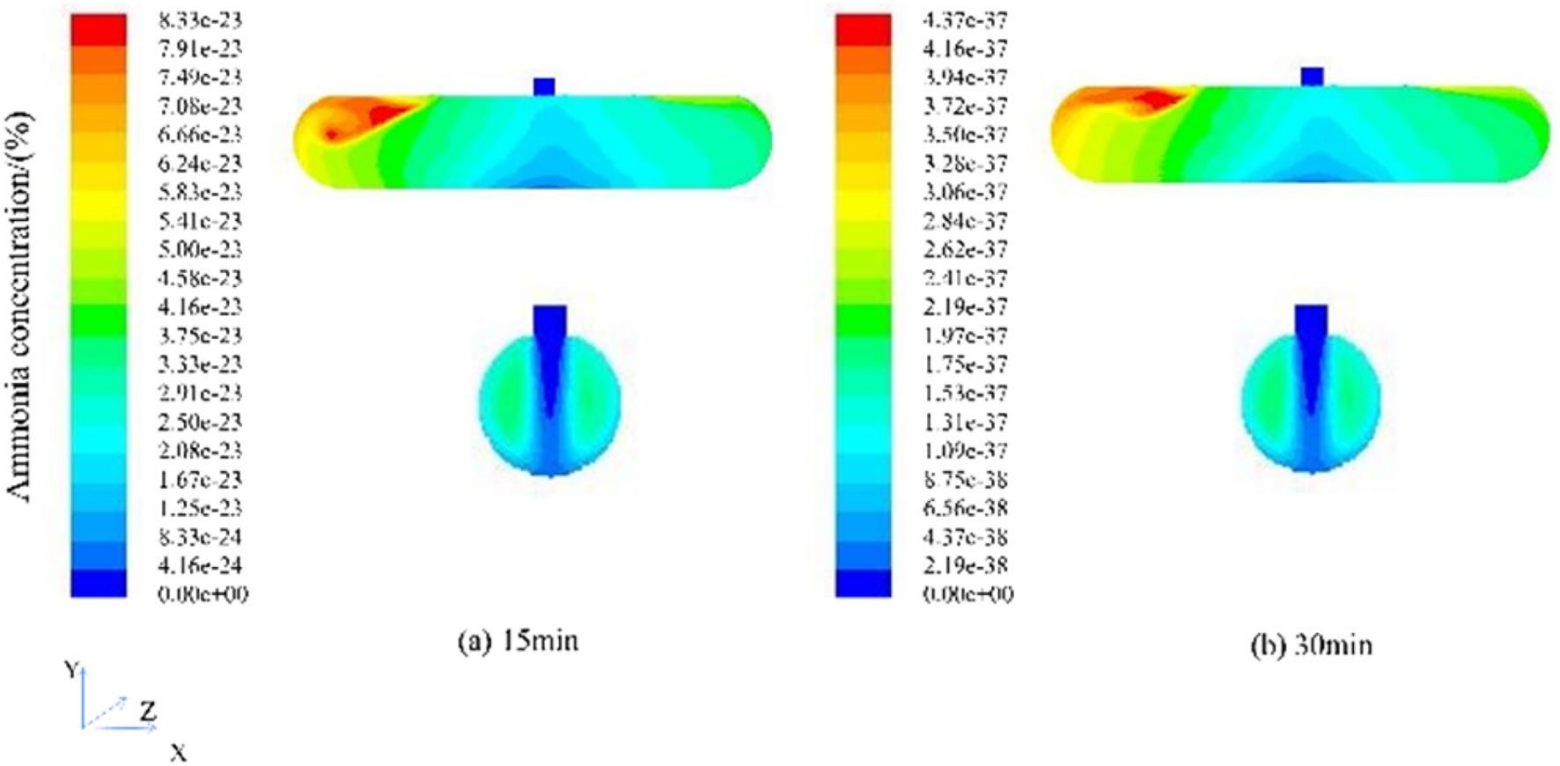
Cloud figure of ammonia concentration distribution at different times (Face and manhole profile).

According to the program of the ammonia gas concentration of the mechanical pressing type, under the condition of the ventilation rate of 10 m/s, the ventilation can be stopped for 2 min. However, in the actual operation process, to prevent the local ammonia concentration from being too high, a 5-min ammonia concentration distribution cloud figure was simulated. After 5 min of ventilation, the highest ammonia concentration in the tank was 1.10e−06, which can be carried out repair work. According to the confined space detection principle, the detection time should not be earlier than 30 min before the start of the operation. Continue the simulation to 30 min, and the obtained ammonia concentration distribution cloud figure, as shown in Fig. 14.

Ventilation to 30 min, the highest concentration of ammonia gas in the tank is 4.37e−37, the concentration is infinitely close to 0, which meets the maintenance conditions and also reaches the principle of maintenance, and can be relatively safe for cleaning and maintenance operations. To ensure the safety of the operators, even if the maintenance standards are met, to prevent the local concentration from being too high in the actual process, the operators should be equipped with operation equipment that has passed the quality inspection.

Due to the mechanical ventilation rate set at the manhole being 10 m/s, the rate remains constant throughout the entire simulation process. The overall wind speed change can be measured through monitoring points. Similarly, as shown in Fig. 12, under this ventilation condition of mechanical ventilation, the wind speed fluctuation at each detection point is relatively small, and the overall wind speed is relatively large. Although there are drawbacks in the dead corner area of air supply, where fresh air cannot effectively flow inside the tank and is not conducive to diluting the toxic gases in the entire area of the tank, the overall ventilation efficiency is much higher than natural ventilation.

Through the simulation of ventilation under different methods, the results show that mechanical ventilation can reach the ammonia concentration approaching 0 in 30 min, while natural ventilation can reduce the ammonia concentration to the safe range in 48 h. The efficiency of mechanical ventilation is much higher than that of natural ventilation. Compared with 0°, the ventilation rate is slightly faster at 45°, but the overall ventilation time required is 48 h. Compared with the mechanical ventilation type, the ventilation rate of the mechanical ventilation type is slightly faster, but the overall ventilation time required is 30 min. According to the ammonia concentration cloud map, the ammonia concentration at the manhole of the horizontal tank is low, and the ventilation efficiency at both ends of the tank is not high, which is easy to cause local ammonia accumulation. In the process of extracting ammonia, the ammonia concentration in the upper part of the tank is higher than that in other places, which is easy to cause harm to operators.

## Simulation results verification analysis

### Validation and analysis of natural ventilation results

In order to verify the reliability of the above simulation results, the method of reference^41^ is used to theoretically calculate the change of ammonia concentration in the horizontal tank under natural ventilation. In the oxygen profit-and-loss game problem, ammonia leakage and fresh air replenishment occur at the same time, and they contribute to the decrease and increase of the oxygen volume fraction respectively, which are two opposite processes. Based on this, it can be considered that the oxygen gain and loss process is due to the “squeeze” or “exit” of ammonia, which leads to a decrease or increase in the proportion of oxygen, so the overall integral C of ammonia and nitrogen in the air is selected as a direct indicator^41^.

At the same time, considering the influence of ammonia leakage and ventilation on the overall nitrogen and ammonia gas fraction, assuming that the normal volume fraction of nitrogen in the air is C_0_, after a time ΔV, the nitrogen and ammonia gas overall fraction increases from C_0_ to C, and nitrogen and ammonia gas The total volume change of is ΔV, according to the law of conservation of volume, the formula (Yin 2022) can be deduced:

9 $$\Delta V = {\text{q}}\Delta t + C_{0} Q\Delta t - C(q + Q)\Delta t$$

In the formula: Δt is an infinitely short time, h; ΔV is the total volume change of nitrogen and ammonia during Δt, m^3^; q is the leakage flow of ammonia gas, m^3^/h; Q is the fresh air volume, m^3^/h; C_0_ is the fresh air The volume fraction of nitrogen in the middle, %; C is the total fraction of nitrogen and ammonia gas, %; (q + Q) is the flow rate of the gas discharged to the outside, m^3^/h. Divide both sides of this formula by the volume Vr, and arrange and integrate to get^41^:

10 $$\int\limits_{{C{}_{1}}}^{{C_{2} }} {\frac{{V{\text{r}}}}{{(q + C_{0} Q) - (q + Q)C}}dc = \int\limits_{{t_{1} }}^{{t_{2} }} {dt} }$$

11 $$t_{{2}} - t_{1} = - \frac{Vr}{{Q + q}}\ln \frac{{(q + C_{0} Q) - C_{1} (q + Q)}}{{(q + C_{0} Q) - C2(q + Q)}}$$

In order to facilitate the analysis, Q = 0 is now set, that is, there is no obvious ventilation process inside the tank, then the formula (11) is simplified to^41^:

12 $$t_{2} - t_{1} = \frac{Vr}{q}\ln \frac{{1 - C_{1} }}{{1 - C_{2} }}$$

In the formula: C_1_ is the initial volume fraction of nitrogen in the air, corresponding to the initial state when the oxygen concentration drops, %; C_2_ is the total nitrogen and argon integral fraction corresponding to the oxygen concentration dropping to the asphyxiating concentration, %. The volume of the horizontal tank is 57.11 m^3^. Assuming that the oxygen volume fraction in the initial air is 20.8%, the flow rate of ammonia leakage is 1.6 m^3^/h, and the oxygen concentration gradually decreases to 18% (anoxic concentration), the overall nitrogen and ammonia integral fraction is 78.2% rises to 81%,bring into formula (12) and calculate t_2_ = 49.25 h. Through theoretical calculation, under the condition of natural ventilation, it takes 49.25 h to reduce the ammonia concentration in the tank to the safe range of 0.0127 mg/m^3^, which is 1.25 h different from the simulation result, indicating that the simulation result has a certain reference value, which can be used in the text The simulation method obtains the change of ammonia gas concentration under natural ventilation, which provides a basis for the safety of actual tank maintenance and other operations.

### Validation and Analysis of Mechanical Ventilation Results

Under the condition of mechanical ventilation, the fan produces a large amount of air, so the ventilation time is much shorter than that of natural ventilation. The ventilation times of confined space operations and accidents should be greater than or equal to 12 times/h^42^, according to formula^43^:

13 $$L = N \times V$$

**Figure 15:**
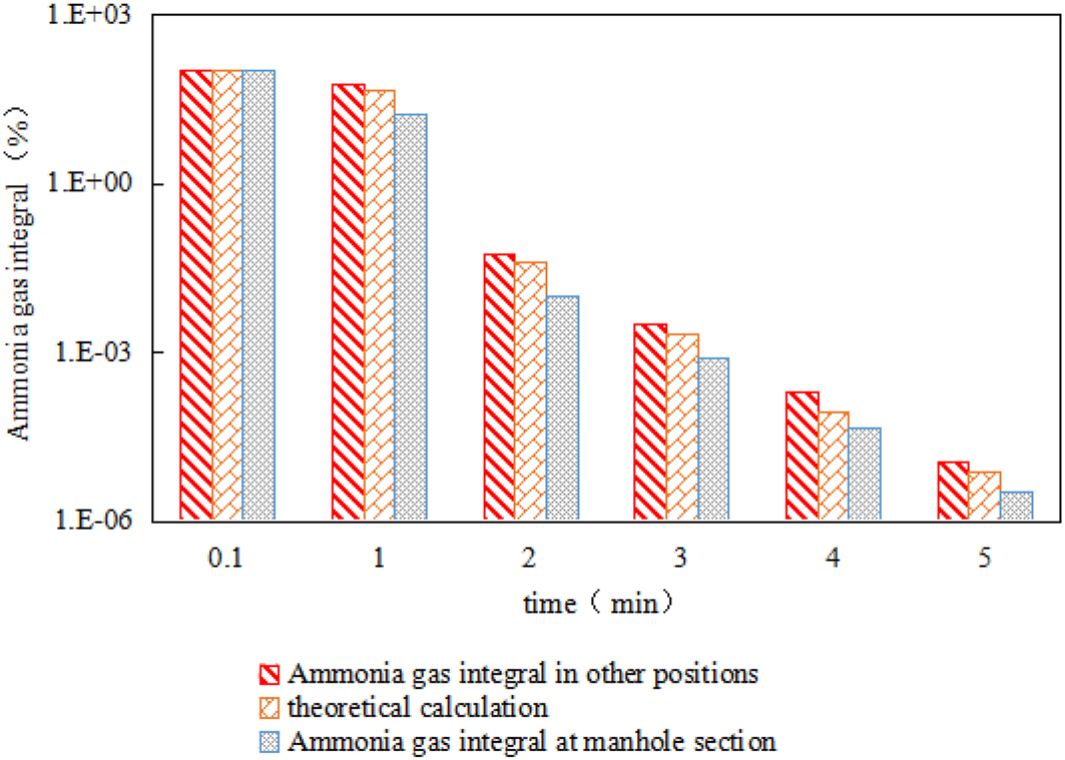
Comparison of ammonia concentrations at different locations.

In the formula: L is the air volume, N is the number of air changes, and V is the volume. From the above conditions, the number of ventilation times of 12 times is multiplied by the volume of the horizontal tank 57.11 m^3^, and the ventilation rate under mechanical ventilation is 685.32 m^3^/h, and the calculated ventilation rate is brought into the formula (12) to calculate t_2_ = 0.11 h, that is, 6.9 min can reduce the ammonia concentration in the tank to the safe range of 0.0127 mg/m^3^, which is 4.9 min different from the simulation result. The mechanical ventilation is also regarded as the process of ammonia gas leakage. Under the condition of known 30 min duration, the total integral of nitrogen and ammonia is calculated from 78.2% to 99.9% by entering formula (12), and the ammonia gas in the tank is discharged. The ammonia concentration can be reduced to close to 0 within the specified 30 min. Bring the time into formula (12) to calculate the ammonia concentration at different times, and compare the ammonia concentration at the manhole and other positions (taking the tank wall on the right side as an example) for mechanical ventilation, as shown in Fig. 15. The theoretical calculation has the same trend as the simulation results, and the average concentration of the entire tank can be obtained, but the specific ammonia concentration distribution inside the tank cannot be seen. Through the simulation, it can be seen that the ammonia concentration at each location is high and low, indicating that the simulation results have With a certain reference value, the simulation method in this paper can be used to obtain the change of ammonia gas concentration under natural ventilation, which can provide a basis for the safety of actual tank maintenance and other operations.

It can be seen intuitively from the histogram chart that when the abscissa time is the same, the ammonia concentration at the other positions is slightly higher than that at the manhole; when the ammonia concentration is the same, the ammonia at the manhole is higher than that at the other positions. After calculation, the reduction rate of ammonia gas at the manhole is 8.3% every 1 min, and after 3 min due to the low concentration of ammonia gas, the change rate is reduced, and the reduction rate of ammonia gas at other positions is 6.1% every 1 min. Therefore, the gas concentration variation gradient near the manhole is larger.

**Figure 16:**
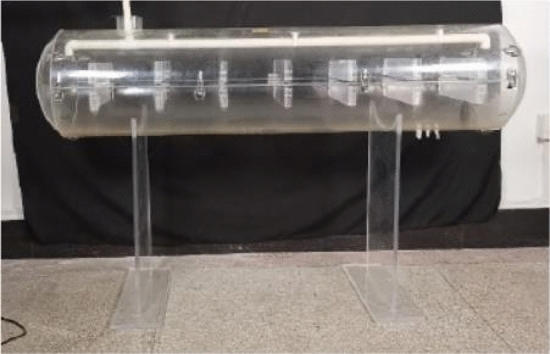
Ventilation experimental device^44^.

Based on the numerical simulation analysis of the vehicle mounted tank mentioned above, it is concluded that the ventilation effect of horizontal tanks with limited inlet and outlet is poor, and the ventilation time is long, which leads to workers not effectively ventilated the tank before entering the confined space for inspection and maintenance. There is residual toxic gas in the internal space, and the oxygen content in the tank is low, resulting in personnel poisoning and suffocation. Due to limitations in the resources and conditions of ventilation experiments, reference^44^ can determine the required ventilation duration for similar tank bodies, in order to validate the effectiveness of the simulation again. The main body of the ventilation experiment is a cylindrical tank, and the experimental material is made of transparent acrylic material, which can visually observe the changes in the ventilation effect inside the tank^44^. The experimental setup is shown in Fig. 16.

The main goal of horizontal tank ventilation is to quickly and efficiently dilute toxic gases inside the tank, but ventilation can cause differences in the dilution degree of harmful gases in different areas of the tank. Therefore, to evaluate the effectiveness of diluting harmful gases inside the tank under different ventilation conditions, a comparative analysis should be conducted based on the overall dilution efficiency and uniformity of the harmful gases inside the tank after the same ventilation time. The higher the relative dilution rate and the more uniform the dilution degree, The better the ventilation effect^44^. Post process the gas mass fraction monitoring data set in Fluent to obtain the remaining harmful gas concentration M1 in the tank after ventilation under corresponding working conditions. The parameter K is defined as the relative dilution rate of harmful gases, which represents the degree to which the concentration of harmful gases in the tank decreases relatively after 100 s of ventilation. The larger the relative dilution rate K value, the faster the ventilation rate, and the better the ventilation effect; The smaller the K value, the slower the ventilation rate and the worse the ventilation effect^44^; The calculation formula is ^44^:

14 $${\text{K}} = \frac{{M_{0} - M_{1} }}{{M_{0} }}$$

In the formula: M_0_ initial concentration of harmful gas; M_1_Residual concentration of harmful gas after ventilation.

Revise the formula and introduce parameters ƞ Denotes the non-uniformity coefficient of gas dilution in each area of the tank after ventilation is completed, and the non-uniformity coefficient ƞ The larger the value, the more uneven the degree of dilution of the gas; Uneven coefficient ƞ The smaller the value, the more evenly the gas is diluted, and parameters are introduced into each formula θ, To correct the differences between horizontal tanks in the literature and simulated horizontal tanks, the relevant formula is as follows^44^:

15 $$\overline{M}{\prime} = \frac{{\mathop \sum \nolimits_{i = 1}^{n} M_{i}^{\prime} }}{n} + \theta$$

16 $$\sigma^{\prime} = \sqrt {\frac{{\mathop \sum \nolimits_{i = 1}^{n} \left( {M_{i}^{\prime} - \overline{{ M_{i}^{\prime } }} } \right)^{2} }}{n}} + \theta$$

17 $$\eta = \frac{\sigma ^{\prime}}{{\overline{M^{\prime}} }} +$$

In the formula: $M_{i}^{\prime}$ reduction of ammonia in various areas of the tank after ventilation; $\overline{{M }^{\prime}}$ The average concentration of the diluted ammonia; n Number of ammonia concentration detection points; ${\sigma }^{\prime}$ Mean square deviation; ƞ The non-uniformity coefficient of the diluted ammonia; θ Correction factor.

**Figure 17:**
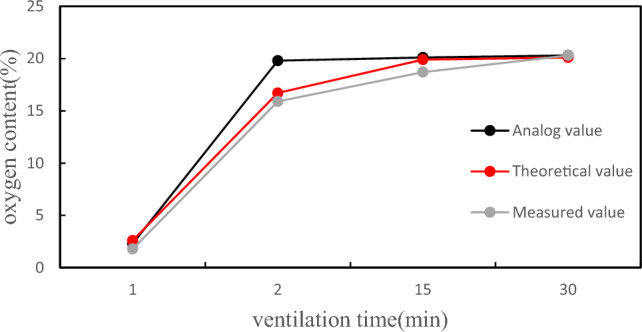
Comparison of analog, theoretical, and measured values.

Numerical simulation is conducted on the conventional ventilation method of vertical tanks, that is, direct air supply at the manhole, and the non-uniform coefficient of wind speed under the basic working condition is calculated through post-processing ƞ Is 3.56, the relative dilution rate K of the ventilation gas is 19.8%, and the non-uniformity coefficient of the diluted gas ƞ 1.75 indicates that under conventional ventilation conditions, there are drawbacks such as low and uneven ventilation efficiency, and harmful gases being limited to one end of the tank body. Under mechanical ventilation conditions, the simulated values obtained were compared and analyzed with theoretical and experimental test values, and the results are shown in Fig. 17.

By comparing the data, it can be seen that the simulation reached the qualified oxygen concentration earlier than in theory and experiment, and it was reached after 2 min of ventilation. However, at this time, both the theoretical and measured values did not reach the qualified value; After 15 min, except for the measured values, the oxygen concentration has been qualified; After 30 min, the oxygen concentration stabilizes at around 20% and can enter the tank for maintenance and other operations. Due to differences in parameter settings and experimental environments during the simulation and experimental processes, there is a slight deviation in the ventilation results, but the overall trend is similar. Therefore, the simulation results and theoretical analysis can be used to verify and risk analyze the tank body.

## Discussion of ventilation results

In this paper, different ventilation methods are used to simulate the ventilation effect and ventilation time of the horizontal ammonia tank, and the ventilation efficiency of the horizontal ammonia tank under four different ventilation conditions is studied. Transport characteristics inside the tank. The results show that it takes 30 min for mechanical ventilation to reach the ammonia concentration that tends to 0, while it takes 48 h for natural ventilation to reduce the ammonia concentration to within a safe range. The efficiency of mechanical ventilation is much higher than that of natural ventilation; natural ventilation is 45° Compared with 0°, the ventilation rate is slightly faster at 45°, but the overall required ventilation time is 48 h; compared with the mechanical ventilation extraction type and the press-in type, the ventilation rate of the press-in type is slightly faster, but the overall ventilation time is slightly faster. The required ventilation time is 30 min; according to the concentration cloud figure of ammonia gas, the ammonia gas concentration at the manhole of the horizontal tank is relatively low, and the local ammonia gas accumulation is likely to occur at both ends of the tank due to the low ventilation efficiency. Among them, the ammonia concentration in the upper part of the tank is higher than in other positions in the process of extracting ammonia gas by mechanical ventilation, which is easy to cause harm to operators. However, in the actual process, the wind direction of the natural wind will change, and it will not always remain unchanged at 0° and 45°; the difference in grid division in the early stage of the simulation will also have a certain impact on the simulation. Subsequent research to strengthen the influence of different grid divisions on the simulation results, numerical simulation, and experimental methods will also be used to study the ventilation results more accurately.

**Figure 18:**
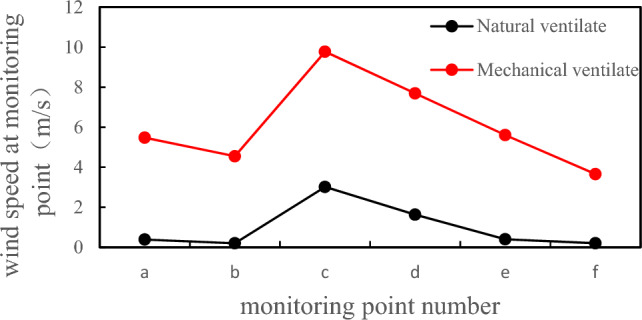
Comparison of wind speeds between natural ventilation and mechanical ventilation.

Compared with natural ventilation and mechanical ventilation, the biggest difference lies in the different ways of taking fresh air. Natural ventilation is to realize the intake and exhaust of fresh air naturally, and mechanical ventilation refers to the use of electrical equipment such as fans and fans to realize the intake and exhaust of fresh air^45^. Natural ventilation is convenient and fast, but the efficiency is not high. Mechanical ventilation can achieve the best ventilation effect in the shortest time with the help of external force. The ventilation rate of mechanical ventilation is higher, and the air disturbance caused by natural ventilation is more intense at the same time, and the diffusion effect of ammonia gas molecules is more obvious, while the diffusion effect of natural ventilation is very weak^46^. Comparing the average speed of natural ventilation and mechanical ventilation monitoring points, as shown in Fig. 18, it is evident that the speed of mechanical ventilation is 3–5 times that of natural ventilation. At point c, i.e. the manhole, the speed of mechanical ventilation is 7 times that of natural ventilation. Therefore, choosing mechanical ventilation is more conducive to the ventilation and maintenance work of the tank body, and it is also an optimization of conventional natural ventilation.

Compared with natural ventilation at 0° and 45°, the biggest difference lies in the difference in wind direction. The horizontal incoming air will be subject to a certain resistance at the only vent manhole of the horizontal tank. According to Bernoulli's equation, the wind speed will decrease, and at the same time, the air volume of the horizontal incoming air entering the manhole will be less, which will eventually reduce the ventilation efficiency. The wind angle of the incoming wind at 45° deviates to a certain extent, and it will also be hindered at the manhole, but the wind resistance generated is smaller than that of the horizontal incoming wind. The air volume of the incoming air has increased, so the efficiency of ventilation is slightly improved. However, because the set external wind speed is low, the wind speed entering the tank is even lower, so the ventilation time has not been significantly improved, and it takes 48 h.

Compared with the press-in type, the biggest difference between the mechanical ventilation draw-out type and the press-in type is the difference in principle. Extraction ventilation draws out the ammonia gas in the tank by an external force, the external air leakage is small and easy to manage, but it is easy to cause the influx of external harmful gases and the power consumption is large; Oxygen and nitrogen) are flushed into the tank to reduce the concentration of ammonia gas, resulting in less external leakage of harmful gases and less power consumption^47^. According to the cloud figure of ammonia gas concentration, the ammonia gas concentration of the mechanical extraction type is easier to accumulate on the entire upper end of the tank. This is related to the extraction method and factors such as the density of ammonia gas is lighter than that of air. The left and right sides of the upper end are piled up.

Although the influence factors such as wind speed and direction are taken into account as much as possible in the selection of numerical simulation conditions in this paper, there are still certain errors. The reasons for the errors are: (1) The wind direction and wind speed will change under actual natural ventilation conditions; (2) Generally speaking, the wind has a certain inclination angle, and relative to the horizontal direction, the general variation range of the wind inclination angle is − 10° ~  + 10°^40^; (3) During the numerical simulation process, the simulation results will vary with the mesh size^48^. Theoretically speaking, when the boundary conditions are set correctly, the higher the element order, the greater the mesh density, and the more accurate the calculation results, but at the same time, the time cost is the same as more computer resources^49^. Although there is a certain error, the density of the mesh is increased, which makes the simulation results reliable. The simulation also provides a theoretical basis for the actual ventilation time. Through the cloud figure of ammonia gas migration inside the tank, it is easier to identify the location where the local concentration is too high so that the staff can enter the ammonia gas tank for maintenance and reduce the risk practical meaning. In practical research and scene application, mechanical ventilation is chosen in most cases because of its higher efficiency and better ventilation quality. However, in this study, only quantitative comparison of various ventilation differences exists certain limitations, in the subsequent research, the ventilation of the tank can be optimized, and mixed ventilation can be adopted to solve the problem of excessive local concentration in the tank.

## Conclusion

1. Compared with mechanical ventilation, mechanical ventilation improves the ventilation rate with the help of the power of the axial flow fan, and the efficiency is higher. The ammonia concentration can be reduced to a safe range within the specified 30 min, while the natural ventilation cannot be used within 30 min. The ammonia concentration is reduced to a safe range, and the highest concentration reaches the safe range after 48 h of simulation, which requires a long time and low efficiency.
2. Compared with the natural ventilation of 0° and the natural ventilation of 45°, according to the cloud figure of the concentration distribution of ammonia gas, at the same ventilation time, the average ammonia gas concentration in the tank is lower when the wind is 45°, and the diffusion of ammonia gas faster. During the simulation process, the ammonia gas on both sides of the tank is not easy to be discharged and is easy to accumulate, which poses a high risk to the operators who enter the tank.
3. Compared with the mechanical ventilation press-in type, the ammonia diffusion rate of the press-in type is slightly higher than that of the extraction type; The height is mainly on the upper part of the tank, which has a higher risk to the operators entering the tank. The overall concentration of the press-in type will be lower and the safety will be higher.

## Data Availability

All data generated or analysed during this study are included in this published article.
